# Comparing meat and meat alternatives: an analysis of nutrient quality in five European countries

**DOI:** 10.1017/S1368980023001945

**Published:** 2023-12

**Authors:** Thies Petersen, Stefan Hirsch

**Affiliations:** 1 Department of Management in Agribusiness (410C), Institute of Farm Management, University of Hohenheim, Stuttgart, Germany; 2 Professorship Agricultural and Food Economics, TUM School of Management, Technical University of Munich, Munich, Germany

**Keywords:** Meat substitute, Red meat, Poultry meat, Nutritional composition, Nutrients, Nutritional comparison

## Abstract

**Objective:**

To assess and compare the (macro-)nutritional composition of red meat (RM) and poultry meat (PM) products with the emerging category of meat substitutes.

**Design:**

We use information on nutritional values per 100 g to estimate the differences in the nutritional composition between RM, PM, vegan meat substitute (VMS) and non-vegan meat substitute (NVMS) and derive six unique meat product clusters to enhance the comparability.

**Setting:**

Meat markets from five major European countries: France, Germany, UK, Italy and Spain.

**Participants/Data:**

Product innovation data for 19 941 products from Mintel’s Global New Product Database from 2010 to 2020.

**Results:**

Most of the innovations in the sample are RM products (55 %), followed by poultry (30 %), VMS (11 %) and NVMS (5 %). RM products exhibit a significantly higher energy content in kcal/100 g as well as fat, saturated fat, protein and salt all in g/100 g than the meatless alternatives, while the latter contain significantly more carbohydrates and fibre than either poultry or RM. However, results differ to a certain degree when products are grouped into more homogeneous clusters like sausages, cold cuts and burgers. This indicates that general conclusions regarding the health effects of substituting meat with plant-based alternatives should only be drawn in relation to comparable products.

**Conclusions:**

Meat substitutes, both vegan and non-vegan, are rated as ultra-processed foods. However, compared with RM products, they and also poultry products both can provide a diet that contains fewer nutrients-to-limit, like salt and saturated fats.

Meat provides a dense form of valuable macro- and micronutrients, but its environmental impact, for example, carbon footprint, is worse than that of alternative plant-based protein sources such as peas^(1,2)^. Furthermore, there is growing ethical concern among consumers about production methods within the meat industry and animal welfare^(3)^. Finally, ongoing research indicates that overconsumption of meat, particularly processed meat products, is associated with detrimental effects on health and increasing the incidence of non-communicable diseases (NCD), such as hypertension^(4,5)^. The food industry has responded to these objections to meat products by developing meat substitutes. Meat substitutes are defined in this paper as products that mimic the taste, appearance, texture and smell of meat products such as steak or salami^(6)^. In addition, we consider products such as tofu as meat substitutes because they replace the function of meat in a meal. In contrast, we do not consider products such as cheese, insects, peas or fish as meat substitutes^(6)^. Although their environmental impact is lower^(7)^, meat substitutes are also considered unhealthy because most of them can be classified as ultra-processed foods^(8)^. These are, like processed and red meats (RM), associated with detrimental health effects^(9)^. For example, higher shares of calories originating from ultra-processed foods as classified by the NOVA system^(8)^ in diets are, similar to meat, associated with adverse effects on cardiovascular health^(9)^. In this context, a study based on a large sample (*n* 21 212) for the French market found a positive correlation between a higher avoidance of animal products in diets and the consumption of ultra-processed foods^(10)^. This raises, in terms of public health concerns, the question of whether meat substitutes can improve the nutritional composition of diets compared with conventional meat products^(11)^. Therefore, in this paper, we use a holistic and multinational sample of 19 941 product innovations from five major European countries over a period of 11 years to analyse the differences in the nutrient content of meat substitutes, poultry and RM products.

Although eating habits differ across European countries and regions, high meat consumption is common and usually exceeds the recommendation of the World Cancer Research Fund of 350–500 g/week^(12)^. The UK has the lowest annual consumption among the countries studied, with 71·6 kg/capita, while it is highest in Spain at 105·8 kg/capita^(13,14)^. The annual meat consumption in the European Union (EU) 28 and the sample countries is presented in the online supplementary material, Supplemental Table 1.

The nutritional composition of meat and meat substitutes is a widely debated subject, particularly given the concerns regarding the overconsumption of certain nutrients, for example, saturated fats and salt, found in both processed RM and ultra-processed foods^(8,15)^. RM products, and especially processed meat products, have a high salt content^(16)^. While Na, as part of dietary salt, is an essential nutrient^(17)^, excessive intake is associated with higher blood pressure and consequently a greater risk for CVD^(18)^. Hence, the WHO recommends a maximum salt intake of 5 g/d for adults to reduce the problem of hypertension and other diet-related NCD^(19)^. However, the actual intake of salt in Europe exceeds the amount recommended by the WHO, ranging from 8 to 12 g/d in most European countries^(20)^.

The overconsumption of energy-dense foods is associated with a higher risk of obesity^(21)^, which in turn is a risk factor for several NCD^(22)^. The WHO nutrition recommendation foresees an intake of under 30 % of the total energy supply from fats and under 10 % from saturated fats^(19)^. However, actual consumption in Europe is higher, whereby meat is one of the major sources of total fats and saturated fats^(23)^. In addition, ultra-processed foods are usually characterised by high fat levels per 100 g^(24)^. The risk of CVD can be lowered by using polyunsaturated fats from plant-based products instead of animal-based saturated fats^(25)^.

In Europe, meat is one of the primary sources of high-quality protein^(4,26)^. On the other hand, plant-based proteins are less digestible than those of animal origin^(26)^. However, this can be improved by processing techniques like fermentation or cooking^(27)^. Furthermore, a meta-analysis suggests that the risk of CVD can be reduced by using plant-based proteins instead of animal-based proteins^(28)^. While the WHO recommends for healthy adults, both men and women, a safe intake level of 0·83 g/kg body weight^(29)^, most adults in high-income countries exceed this recommendation^(30)^. Diets with a protein intake that exceeds the recommended amount can be related to a higher risk for type 2 diabetes^(30)^.

The previous literature on differences in the nutritional composition of meats and meat substitutes is limited; it is often based on small sample sizes and presents mixed results. One study comparing the nutrient content of modern meat substitutes and meat products yields inconclusive results regarding which of the two options is healthier from a nutritional viewpoint^(31)^. However, the study size is limited on a sample of just thirteen individual products^(31)^. A second study, carried out in the market of the UK in 2020 involving a total of 207 meat substitutes and 226 RM and poultry meat (PM) products, reported that the nutrient composition of meat substitutes is beneficial, but thereby it does not examine the role of carbohydrates^(32)^. A third study based on 137 products for the Australian market reported mixed results for the differences in nutritional values when comparing the product groups of burger, sausages and minced meats^(33)^. Moreover, a study of the German meat market found based on an aggregated score fewer ‘nutrients to limit’, that is, salt, sugar, saturated fat and energy content, in meat substitutes than in RM and PM products^(6)^. Finally, a recent study of the Italian meat substitute market based on 269 products reported some nutritional benefits of meat substitutes; however, it does not recommend them as a wholesome replacement for meat^(34)^.

Our research contributes to the literature as follows: We use a holistic sample of 19 941 individual products introduced between 2010 and 2020 in five major European countries, France, Germany, UK, Italy and Spain, to compare and analyse the nutritional composition of meat substitutes and traditional meat products. Thereby, we provide data for the nutritional composition of products on a disaggregated scale. And finally, the comparability is enhanced by systematically grouping RM products and PM products and the corresponding meat substitutes into homogenous clusters, like burgers or sausages. To the best of our knowledge, our analysis provides the first holistic comparison of the nutritional characteristics of meat and meat substitutes across European countries. While meat consumption patterns differ perceptibly, there are similarities in the overall level of high meat overconsumption.

## Materials and methods

We used data from Mintel’s Global New Product Database (GNPD)^(35)^. This keeps abreast with the fast-moving consumer goods market and provides information and data about product innovations, which are being launched in supermarkets in countries worldwide. The product data are entered into the database by shoppers and offers a wide range of information about the products, such as the region where the product was introduced, the date of market introduction, the producer, the complete information provided on the product package, including the nutrients and ingredients, plus pictures of the product, its size and price^(35)^. Our initial search for all RM, PM and meat substitutes introduced in the five European countries studied, France, Germany, UK, Italy and Spain over the time frame 2010–2020, resulted in 27 375 product-level observations.

Packaged food products in the EU must comply with legal requirements regarding the information provided on the packages^(36)^. As this includes detailed information on the ingredients and the nutritional values of the products^(36)^, we were able to conduct our nutritional comparison of products based on information for the following nutrients: energy content in kcal/100 g, fat, saturated fats, carbohydrates, sugar, protein, fibre and salt (all in g/100 g). In cases where products indicated the Na content instead of the salt content, we used the molecular weight of Na and chloride to calculate the salt content. Since information on the fibre content is not mandatory on all products, it was calculated based on energy levels. In accordance with the literature^(37)^, the calculation was carried out using the energy levels of protein (4 kcal/g), carbohydrates (4 kcal/g) and fat (9 kcal/g) in the product’s total energy content without fibre. The calculated energy without fibre was then subtracted from the energy level indicated on the package and the number was divided by the energy level of fibre (2 kcal/g). Finally, we replaced the missing fibre values with the calculated values. The conversion factors applied are available in the EU legislation for nutritional information^(36)^. Although this approach is less accurate than an analytical detection of fibre levels, it allowed a larger number of observations to be compared, as only 8598 of the 27 375 products reported the fibre content.

We commenced by checking the minimum and maximum values for each nutrient in each product to identify outliers and incorrect values and thus ensure data accuracy and mitigate potential biases caused by reporting errors in the data. Second, we checked for recording errors in the database, for example, cases in which the saturated fats were reported to be higher than the total fat, which is impossible. In these cases, we used the images of the products to derive the correct values from the nutrition facts label. We corrected a total of 1603 individual values based on the product images. In cases where the information was originally obtained from the product itself, but was obviously incorrect, we excluded the observation from our analysis. Note that this only applied to 155 products (0·5 %). An estimated value for the calories was then calculated based on the information for fat, carbohydrates, fibre and protein, to identify those products with a large, that is, > 10 kcal/100 g, deviation between the estimated value and the caloric value indicated on the package. In these cases, we rechecked to confirm that the information entered in Mintel’s GNPD matched the information on the packages and corrected the value in our database accordingly. In addition, we used STATA’s Bacon algorithm, which is based on Mahalanobis distances, to detect multivariate outliers under consideration of all nutritional indicators and the values calculated for fibre^(38)^. The algorithm detected fifty-one outliers, which were excluded from further analysis. These outliers are characterised by a high divergence between the calculated and the indicated calorie content.

### Meat categories and meat cluster formation

The GNPD database only distinguishes between PM, RM and meat substitute products. However, previous literature has shown that there are nutritional differences between vegan meat substitute (VMS) and non-vegan meat substitute (NVMS)^(6)^. Therefore, the ingredients listed for the meat substitutes included in our sample were used to verify whether a product is vegan or not. This resulted in two distinct groups: VMS and NVMS.

Furthermore, as the meat market encompasses a wide range of heterogenous products, from minimally processed fillet to highly processed ham, which are not only consumed in different portion sizes but are also likely to have different nutritional compositions, we applied a clustering mechanism to group products into more homogenous, more comparable product clusters. A study on the UK meat market grouped products into the following six distinct clusters: sausages, burgers, plain poultry, breaded poultry, mince and meatballs and compared the meat alternatives in each cluster with their traditional meat counterparts^(32)^. A second study applied four clusters: burgers, meatballs, ham and nuggets^(31)^. In line with the previous literature, we create six different clusters: burgers, coated meat, cold cuts, meatballs, meat for roasting and cooking, and sausages. These clusters represent major sectors of the total meat market and we believe that they duly reflect its heterogeneity, thus facilitating a better comparison of individual products. We created keywords for each cluster, for example, quarter pounder for burgers, or nuggets for coated meats. Online supplementary material, Supplemental Table 3 presents a sample list of keywords for each cluster, and the full list is available from the authors upon request. We then matched the names of the products with the keywords and assigned the products to the respective cluster. Our aim was to allocate each product in the sample to one specific cluster, but in some cases a product was placed in more than one cluster. One example of this is a product named ‘burger bacon’, which was not only allocated to the burger cluster but, due to the keyword ‘bacon’, was also to be found in the cold cuts cluster. In cases where the name did not tally specifically with one individual cluster, we checked the images of the products and allocated them manually to the best fitting cluster. Products which could not be assigned to one specific cluster were excluded from the whole study. This reduced our sample size by 1582. In addition, we excluded 205 assortments from the analysis, for example, packages containing a variety of different hams. In a last step, products with missing values for nutrients other than fibre were excluded.

### Statistical analysis

The main objective of this study is to compare the nutritional values of products in the market for meat and meat substitutes. Hence, for our estimation, we first assumed that our observations apply to one meat market comprising the four broad product categories, RM, PM, VMS and NVMS products. The differences in nutritional quality between these product groups were determined by estimating a set of eight linear equations with the individual nutrients (sugar, carbohydrates, fat, saturated fats, protein, salt and fibre each in g/100 g) and the energy content (in kcal/100 g) as the dependent variables and the meat categories (RM, PM, NVMS and VMS) are the independent variables. The multi-equation model is defined as follows and the full list of variable definitions is reported in online supplementary material, Supplemental Table 1:

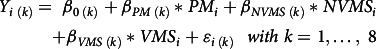

where *i* indicates the 19 941 products and *k* indicates the eight different regressions with the nutrients and calorie content as dependent variables each reflected by *Y*_(*k*)_. *PM*, *NVMS* and *VMS* are dummy variables that take a value of one if product *i* belongs to the respective product category. Finally, *ε*_*i*_ is an error term. In the above model, we used RM as our reference category. We assumed the common null hypothesis for all *β* that the differences in the nutritional values are zero. After estimating each regression model, we compared the *β* coefficients (i.e. the estimated marginal means) for the meat categories pairwise. We corrected the *P*-values for statistical significance with the approach proposed by Benjamini and Hochberg, which corrects for the false discovery rate to avoid an *α* error for the rejection of a true null hypothesis, which can arise by random chance in a multiple comparison context^(39)^. This procedure reduces the risk of a *β* error more effectively than alternative approaches like the Bonferroni correction. In addition, we present the results with the correction proposed by Holm, which corrects for the family-wise error rate^(40)^.

In the second stage of our analysis, we considered the differences between products available in the food market based on the defined clusters of meat product subgroups: burgers, coated meat, cold cuts, meatballs, meat for roasting and cooking, and sausages. We re-estimated the equations defined above for the more homogenous product subgroups and subsequently the estimated marginal means are compared again for each meat category (RM, PM, NVMS and VMS) within the subgroups. Finally, the model was also estimated individually for the different countries to identify regional variations in nutrient quality. The results are presented graphically to illustrate the estimated marginal means and the respective 95 % CI for each nutrient and product subgroup.

## Results

Table 1 presents the cross-tabulation of the meat category and meat cluster distribution. Our total sample of 19 941 products consists of 5·1 % NVMS, 10·5 % VMS, 29·5 % poultry products and 54·9 % RM products. This implies the predominant role of traditional meat products in the market. Roasting/cooking represents the largest product cluster in our analysis (36 % of the total sample) and the meatball cluster is the smallest with 992 products (5 %). Online supplementary material, Supplemental Tables 4–8 show the distributions per country. In general, poultry products play a much greater role in diets in France and the UK^(13,14)^, with shares amounting to 31 % and 39 %, respectively, in the samples, which exceed the shares in Germany, Italy and Spain (23 %, 27 %, 27 %). The share of VMS is lowest in the UK (8 %), while it holds a share of at least 10 % in all other countries. Furthermore, product cluster sizes differ between the countries observed. While the cold-cut category has the highest share in most countries, the roasting/cooking cluster is the largest in France and in the UK.

**Table 1:** Cross table of meat categories and meat clusters across all five countries

Meat category										
Meat cluster	Non-vegan meat substitutes		Poultry meat		Red meat		Vegan meat substitutes		Total sample	
	*n*	%	*n*	%	*n*	%	*n*	%	*n*	%
Number of observations (share cluster of the total meat category)										
Burger	227	22·1	142	2·4	797	7·3	559	26·8	1725	8·7
Coated meat	133	13·0	751	12·8	165	1·5	153	7·3	1202	6·0
Cold cuts	108	10·5	1001	17·0	4671	42·6	113	5·4	5893	29·6
Meatballs	155	15·1	106	1·8	466	4·3	195	9·3	922	4·6
Roasting/cooking	253	24·7	3402	57·9	2557	23·3	874	41·8	7086	35·5
Sausages	149	14·5	473	8·1	2296	21·0	195	9·3	3113	15·6
Total sample	1025		5875		10 952		2089		19 941	
Cat. share of whole sample	5.1%		29.5%		54.9%		10.5%			

Source: Own calculations based on Mintel’s GNPD. Note: Cat. Refers to the meat category.

### Results of pairwise nutrient comparisons across meat categories

Table 2 reports the results of the pairwise comparison for each nutrient across the four broader meat and meat substitute categories. The results for the underlying regressions are reported in online supplementary material, Supplemental Table 10. At 22·5 and 24·3 kcal/100 g, the estimated mean energy content in kcal/100 g of NVMS and VMS is significantly higher (*P* < 0·001) than the energy content of PM. In contrast, both NVMS and VMS have a significantly lower (*P* < 0·001) energy content than RM products, namely 41·2 and 39·4 kcal/100 g, respectively. Finally, while there is no statistically significant difference between the energy contents of NVMS and VMS, RM products have a significantly higher energy content than poultry products.

**Table 2:** Pairwise comparisons of marginal linear predictions of the individual nutrients*

	Energy kcal/100 g		Fat g/100 g		Saturated fat g/100 g		Carbohydrates g/100 g		Sugar g/100 g		Fibre g/100 g		Salt g/100 g		Protein g/100 g	
Comparison	Mean D.†	*P* values††	Mean D.†	*P* values††	Mean D.†	*P* values††	Mean D.†	*P* values††	Mean D.†	*P* values††	Mean D.†	*P* values††	Mean D.†	*P* values††	Mean D.†	*P* values††
Non-vegan MS *v*. poultry meat	22·5	<0·001	1·85	<0·001	−0·47	<0·001	6·63	<0·001	0·89	<0·001	3·31	<0·001	−0·02	0·599	−6·67	<0·001
Non-vegan MS *v*. red meat	−41·2	<0·001	−6·13	<0·001	−4·57	<0·001	10·17	<0·001	1·28	<0·001	3·28	<0·001	−0·92	<0·001	−8·33	<0·001
Non-vegan MS *v*. vegan MS	1·8	0·582	1·22	0·001	0·40	0·004	0·58	0·006	0·08	0·183	−0·64	<0·001	0·15	0·001	−3·52	<0·001
Vegan MS *v*. poultry meat	24·3	<0·001	0·63	0·007	−0·87	<0·001	6·05	<0·001	0·82	<0·001	3·75	<0·001	−0·17	<0·001	−3·15	<0·001
Vegan MS *v*. red meat	−39·4	<0·001	−7·34	<0·001	−4·97	<0·001	9·59	<0·001	1·20	<0·001	3·93	<0·001	−1·07	<0·001	−4·82	<0·001
Red meat *v*. poultry meat	63·7	<0·001	7·97	<0·001	4·11	<0·001	−3·55	<0·001	−0·38	<0·001	−0·18	<0·001	0·90	<0·001	1·66	<0·001

Non-vegan MS, non-vegan meat substitutes; vegan MS, vegan meat substitutes; fibre, calculated fibre content.

* The results are estimated based on the results of the linear models in online supplementary material, Supplemental Table 10.

† Mean D., difference in estimated marginal means between meat categories.

†† The numbers are the Benjamini and Hochberg corrected *P*-values^(39)^.

The fat content in NVMS and VMS is significantly higher than in poultry but lower than in RM products. However, results are different when focus centres on saturated fats, as both meat substitute categories contain, on average, significantly lower levels of saturated fats than RM and PM products. We detected considerable differences when meat substitutes are compared with RM products, with NVMS and VMS undercutting the saturated fat value of RM products by an average of 4·57 and 4·97 g/100 g (*P* < 0·001). We also found that RM contains significantly higher amounts of fat and saturated fat than PM. Finally, differences can also be detected across meat substitutes, with non-vegan alternatives containing on average both significantly more fat and saturated fats per 100 g than VMS.

It can be observed that both types of meat substitutes contain more carbohydrates and sugar than poultry and RM products but, on the other hand, also higher amounts of fibre. Furthermore, VMS contain on average noticeably less carbohydrates and more fibre than NVMS.

Although no statistically significant differences were detected between the salt content of NVMS and PM products (*P* = 0·599), NVMS contain less salt than RM products and VMS contain less salt than either RM or PM products. In fact, VMS also contain on average less salt than non-vegan alternatives. Finally, RM contains significantly higher amounts of salt than PM products.

In general, both types of substitutes have a lower protein content than poultry and RM products (*P* < 0·001). In addition, we found that VMS contain more proteins than non-vegan products (*P* < 0·001) and finally, RM products contain more protein than poultry products (*P* < 0·001). In summary, the pairwise comparison results yield a complex picture of the nutritional differences between emerging meat substitutes and traditional meat products. The implications of these findings are discussed in detail below.

### Results of nutritional comparison based on meat product clusters

**Fig. 1:**
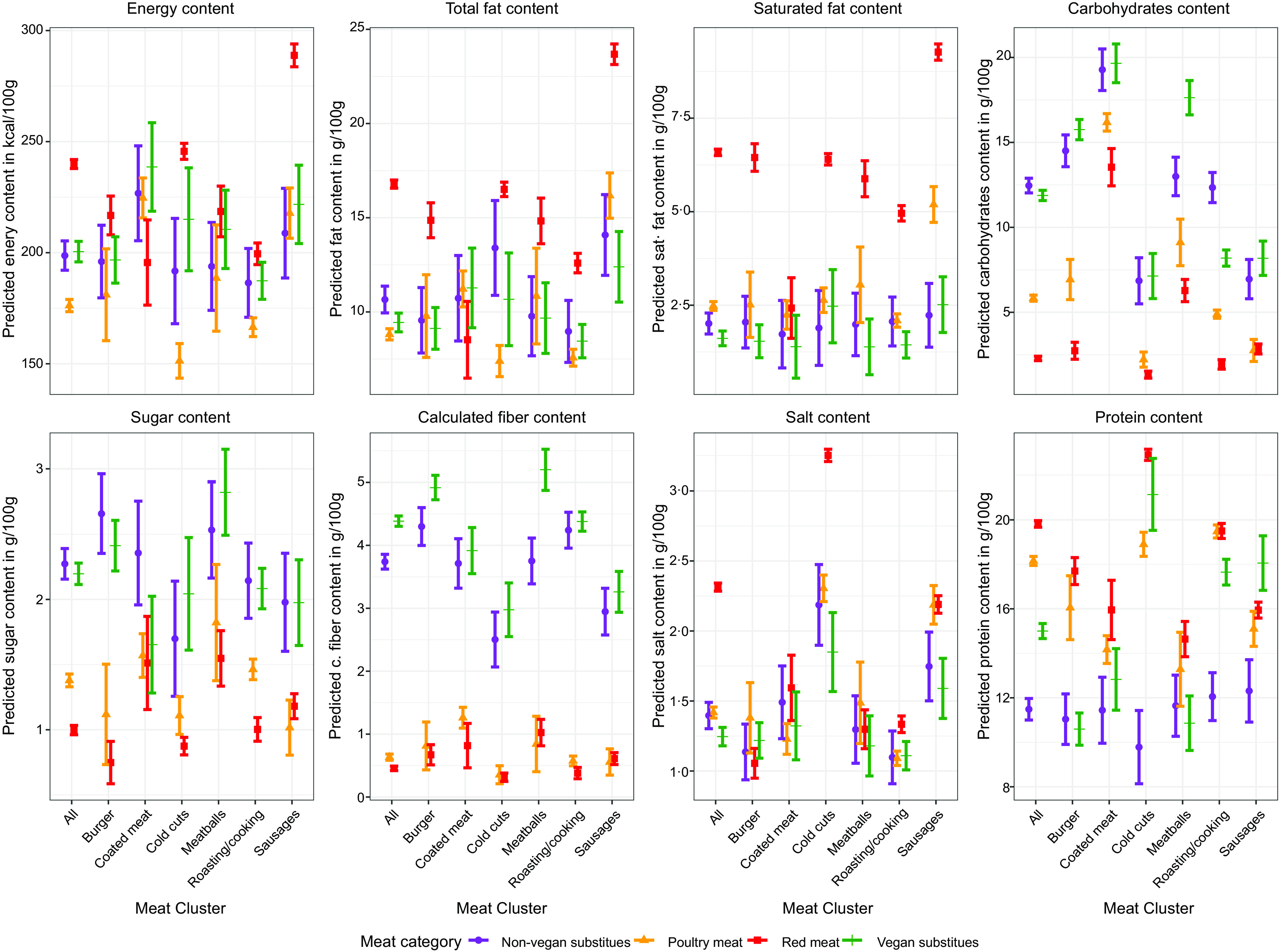
Comparison of predicted marginal mean values with 95% confidence intervals of observed nutrients

The plots in Fig. 1 present the results of the nutritional comparison between the different meat categories (RM, PM, NVMS and VMS) for the individual homogenous meat clusters, that is., burgers, cold cuts and meat balls, etc. To facilitate comparison, the first column of each plot recapitulates the results of the estimations reported in Table 2, that is, relating to the four broader meat and meat substitute categories without consideration of the product clusters. The results confirm that RM products tend to have the highest energy content across individual meat product clusters, apart from the coated meat cluster. In particular, the products in the RM sausages cluster have a considerably higher energy content than any of the other products. In contrast, poultry products tend to have the lowest energy content levels in most clusters, except for coated meat and sausages. To a large extent, these results are due to the high/low fat contents of the respective meat clusters in the RM and PM categories, respectively. The fat content of vegan and non-vegan substitutes differs significantly if product clusters are disregarded, but it does not fluctuate within any of the individual clusters. In general, RM also exhibits the highest levels of saturated fats across the meat clusters, though this is not significantly higher than PM and meat substitutes in the coated meat cluster.

There are only minor amounts of carbohydrates in RM and PM, except for the coated meat and, to some degree, the meatballs cluster. In contrast, the amounts of carbohydrates in vegan and non-vegan meat alternatives are higher across all product clusters and some differences between the carbohydrate content of both meat substitutes can be observed in the roasting/cooking and the meatballs clusters.

A similar picture emerges for the sugar content of the products. While there are only marginal amounts in RM and PM, non-vegan and vegan meat alternatives contain considerably more sugar. However, it must be noted that in view of the WHO recommendation of less than 50 g/d^(19)^, the general sugar levels are relatively low at <3 g/100 g.

Figure 1 shows that there is potential to enhance the amount of dietary fibre by substituting NVMS and VMS for poultry and RM. Moreover, salt intake can be reduced by replacing RM cold cuts with poultry, VMS or NVMS. Finally, VMS and NVMS have a lower protein level with the exception of VMS in the cold cuts and sausage clusters, which exhibit protein levels comparable to RM and PM products.

Online supplementary material, Supplemental Figures 1–4 present the country-wise comparison of the predicted marginal means for the individual product clusters with 95 % CI. The results of the differences in the nutritional composition between the clusters seem to be robust across individual countries. However, it is noticeable that the average salt, energy and saturated fat content of products in the UK is somewhat lower. In addition, the low number of observations within some country clusters leads to a pronounced increase in CI, leading to less significant results between country clusters.

## Discussion

This study, compared the nutritional composition of RM, PM and VMS and NVMS, was carried out against the background of the ongoing discussion of diet-related diseases due to excessive amounts of nutrients-to-limit inherent in meat and ultra-processed products. Based on a sample of 19 941 individual products from the European meat market, we found that RM products are higher in energy, fat, saturated fats and salt than PM and both VMS and NVMS. However, after grouping the products into more homogenous clusters, we found that the high salt content of RM products is a specificity of the cold-cut category. On the other hand, meat substitutes exhibit higher levels of carbohydrates, that is, higher sugar and fibre content than RM and PM. It follows that dietary changes, such as opting for poultry and meat substitutes instead of RM, which is so widely consumed in Europe^(13)^, would reduce the intake of saturated fats, increase the intake of fibre and could potentially lower the incidence of nutrition-related NCD.

Our results indicate significantly higher amounts of saturated fats in RM products compared with PM products and meat substitutes, except for coated meat. For example, at ∼9 g/100 g, sausages contain three times more saturated fats than meat substitutes which represents 41 % of the daily maximum intake recommended by the WHO. Therefore, meat substitutes have great potential in terms of the overall goal of reducing saturated fat intake in the diets, which in turn could reduce the associated detrimental health effects^(25)^.

The overall results indicate that meat substitutes and PM products contain significantly less salt than RM products ranging from 0·9 g/100 g to 1·02 g/100 g of salt. These values indicate that it would be highly recommendable to substitute RM products. However, the results differ somewhat for the individual product clusters. It is noticeable that the average salt content of RM cold cuts exceeds 3 g/100 g of product, which is over 60 % of the WHO recommendation and significantly more salt than found in the other meat categories. On the other hand, the differences and level seem to be less extreme in the burger cluster. Hence, these results underline the importance of considering product clusters when evaluating the health effects of products.

Meat is the major source of protein in Europe^(4)^. Therefore, a high-quality source of protein would be lost if meat was eliminated completely. In general, meat substitutes provide less protein. However, there are large differences between the product clusters and between the VMS and NVMS categories. While both VMS and NVMS contain fewer proteins than poultry and RM in the burger and roasting/cooking clusters, the results are mixed in the other clusters. In addition, VMS in the sausage cluster contain, surprisingly, the highest amounts of protein while NVMS have the lowest protein content. Therefore, it would be interesting to investigate which ingredients drive these protein content results which is beyond the scope of this study.

On average, meat substitutes contain more carbohydrates across all product clusters. We are unable to compare the quality of these carbohydrates as our data only cover the sugar and fibre content. Low-quality carbohydrates might demand a more complex comparison and management of the blood sugar levels for people with diabetes^(41)^. Furthermore, our results regarding the higher fibre content in meat substitutes must be viewed with some caution, as they are not based on an analytical detection of fibre levels reported on the product packages, but on the manually calculated fibre content per 100 g. However, the results do suggest higher amounts of fibre in meat substitutes than in RM and poultry products. Therefore, given the higher amounts of fibre inherent in meat substitutes they can potentially reduce the risks for some NCD if they are used to replace RM products^(41)^.

Based on the NOVA classification, meat substitutes are mainly rated as ultra-processed foods^(8)^. Additionally, consumers use the degree of processing as heuristic to evaluate the healthiness of foods^(42)^. However, when the UK nutritional profiling system is used, not all products classified as ultra-processed foods are rated as unhealthy^(43)^. Therefore, it might be more appropriate to evaluate the healthiness of products based on a detailed product cluster rather than on a processing level, thereby allowing for marginal improvements in nutrient uptake. For example, this could be achieved by replacing RM sausages or cold cuts with poultry or meat substitute counterparts to reduce salt and saturated fat intake. This means that the adoption of mandatory food labelling schemes, like the Nutri-Score, that allow consumers to compare the products within a specific cluster could promote healthier choices.

Traditional vegetarian diets consist of high shares of foods that are not highly processed, like legumes and vegetables^(15)^. However, meat substitutes which conform to a vegetarian diet are highly processed products^(15)^. Although our results suggest that meat substitutes contain lower levels of saturated fats and salt than RM products, they are still likely to contain higher levels of these nutrients-to-limit than unprocessed vegetables. In any case, meat substitutes are probably of little relevance to traditional vegetarians for which a switch to meat substitutes might imply an increased intake of salt and saturated fats. However, most people in European countries include meat in their diets. Therefore, public health outcomes could benefit from a (partial) switch from the consumption of traditional RM products to novel highly processed meat substitutes.

While many aspects of this study are sound, such as the comprehensive product sample, it also has limitations. First, our sample is based on products which are sold in supermarkets, and therefore it provides no information about meat products sold at other points of sale, such as butcher’s shops. However, most of the meat consumed, for example in Germany, is sold in discounters and supermarkets^(13)^. Based on this fact and the large sample size involved, we assume that it is reasonable to draw conclusions about the population of meat products available on the markets analysed. Second, even though the clustering mechanism is based on previous literature^(31–33)^, there might well be more appropriate clusters to distinguish between the different meat products, for example, a salami cluster or a minced meat cluster. However, our main goal was to explore the nutritional differences between meat and meat substitutes and our clustering mechanism adequately fulfils this purpose. Additionally, our research is based on nutritional values only and thus neglects the role of important micronutrients, for example, Fe, Zn and vitamin B_12_ although meat is an important source of these nutrients^(44)^. While plant-based diets can meet the requirements for micronutrient intake, this requires a higher level of food knowledge^(45)^. Therefore, for low-income and lower-educated groups, meat may be an easier way to meet the needs for these important micronutrients^(45)^. Thereby, although the excessive intake of certain nutrients, like saturated fats, is associated with NCD^(25)^, it is important to consider the food matrix of products as well. Foods having the identical nutritional compositions of macronutrients but with different food matrices could react differently during digestion^(46,47)^. Furthermore, the data did not allow us to consider whether a product is sold in more than one country, hence getting more weight in the overall analysis. Additionally, the data did not allow to assess the actual product sales and consumption patterns in the countries we investigated. Hence, it was not possible to derive the true uptakes of nutrients in the population based on the products weighted according to their actual sales. However, our study does allow us to assess the desirability of substituting certain products. For example, the intake of salt and/or saturated fats, which are both associated with detrimental health effects when overconsumed^(18,23)^, could be reduced by replacing RM-based cold cuts and sausages with cold cuts and sausages from other sources. Finally, our study only focuses on the nutritional viewpoint of the meat and meat substitute debate. Thereby, it neglects the ethical aspects of meat production as well as the role of meat in the context of environmental sustainability. Thereby, meat consumption levels in high-income countries have a strong negative external effect on the environment^(48)^. Though there is research on the environmental sustainability of meat substitutes, such as a study on pea-based meat substitutes finding them to have a lower environmental footprint per nutrient than beef^(2)^, future research is needed to assess the environmental perspective holistically.

In view of the rising incidence of diet-related NCD^(49)^, the results presented here should motivate policy makers to support strategies designed to increase the share of poultry products and meat substitutes in consumers’ diets. Although the majority of the latter can be rated as ultra-processed foods, meat substitutes in cold cuts and sausages exhibit lower levels of salt, generally lower levels of saturated fats and a lower energy density while still providing adequate protein levels and significantly more fibre. Hence, the promotion of these meat alternatives could then lead to reduced public health costs by preventing diet-related NCD. Furthermore, the promotion of meat substitutes could generate additional positive effects in European countries with intensive RM consumption by helping to reduce the associated carbon footprints.

## Supplementary material

For supplementary material accompanying this paper visit https://doi.org/10.1017/S1368980023001945
